# New York Letter

**Published:** 1879-09

**Authors:** 


### Domestic Correspondence.

ARTICLE XIV.

NEW YORK CITY.

*Editors* CHICAGO MEDICAL JOURNAL AND EXAMINER:

In the city here just now there is a condition of repose, medically speaking, which makes letter-writing a not very easy matter. Our eminent men have gone to Europe to get rest and new ideas, or to watering places in this country, where they can gather in the occasional shekel at fashionable hotels. This latter practice a good many of our younger men also have embarked in, and many of them do more business in the summer than winter months. In every hotel, amongst the places about the city, there is seen a neat placard in the office announcing that Dr. Such-an-one, of New York, is resident physician. All this to the great pecuniary depletion of afflicted guests and to the grief of the local practitioners.

Those of us who remain in the city, meanwhile, divide our time between controlling diarrhœas and fighting the very elevated temperature with such antipyretic measures as steamboat excursions and cooling drinks.

We have had a little interesting reading furnished us by a polemic upon the caliber of the urethra between Drs. Sands and Otis. Dr. Sands began, I believe, by asserting in a clinical lecture that the theory advanced by Dr. Otis in regard to reflex spasms of the urethra was both untrue and unsafe.

Dr. Otis had asserted that many, if not most deep strictures were due to a slight stricture in the anterior portion of the canal which by reflex action caused spasm further back. He claimed that much harm and unnecessary cutting was done by operating on the deeper portions, where the root of the trouble lay near the meatus, which part should be slit up and dilated freely. Dr. Sands, on the other hand, denied the truth of this and ridiculed the tremendous lacerations which this very innocent portion of the organ was too often subjected to, instancing cases also where he had seen the meatus cut so freely that the stream of urine could only dribble from it. Various other points were brought up also in the discussion, and each eminent gentleman in turn impeached the veracity, courtesy and surgical attainments of the other. It is difficult to say which came out ahead. Certainly nothing very definite or valuable was given to the medical public, and we were only furnished the not very rare spectacle of a couple of surgical brethren bumping their heads together and each calling attention to the very hollow sound that came from the cranial cavity of the other. Dr. Otis has made a valuable contribution to urethral surgery in his having shown the real caliber of the urethra and the frequency and importance of strictures at the meatus. When he gets an idea in his head, however, he makes a good deal of thunder over it, and the present one requires less discussion and more clinical observation before it can be considered proved.

Much of the medical energies of New York are directed in summer towards the hygienic condition of the city, and we are likely in time to become quite a model as regards sanitation and within the limits that our mode of living allows. Every year a corps of visiting physicians to the tenement houses is appointed and they are sent around to hunt up sick children and give them paregoric and chalk mixture, supplying them also with tickets to a free excursion on the river. When the work is faithfully done, and most of it is so performed, there is no doubt of the good results. There is an astonishing amount of lethargy and indifference amongst many of the poor when their children get sick. There are people who will let their children go until just on the point of death rather than take the trouble to call in a dispensary physician not two blocks away. When, however, a physician calls of his own accord and gives them medicine and an opportunity to take a sail in the bay with the babies, they will gratefully accept it.

A new move also has recently been started by those interested in tenement house reform. Forty inspectors have been appointed who are to visit all of the twenty-five thousand tenement houses in the city and take careful notes of every detail concerning them. When this work is finished the board of health will have a complete record of the sanitary condition of every tenement house in the city, sewerage, light, cubic feet of air, etc. It will be invaluable as the basis for future work in the direction of reform amongst these places.

We have had three or four cases of yellow fever here, but no one has been alarmed. Some of the cases showed, however, that the capacity of the yellow fever germ for defying precedent and evading pathological laws is still unexhausted. The bark Wallace left Havanna June 28th, and reached quarantine July 3d, with two cases of malarial fever on board. She was quarantined for ten days and thoroughly disinfected. By the end of that time the sick seamen were well and the vessel proceeded to the city. About July 26th the stewardess was taken ill with symptoms of malarial fever and was sent to Presbyterian Hospital ; two or three days later she died, and just before death the symptoms becoming suspicious, a post-mortem was made and the evidences of yellow fever were found. The incubation of the disease was therefore nearly thirty days, unless it is assumed that the poison was carried about in her clothes for twenty-five days and that it then "struck in." In any event the case is perplexing. Nor is this the end of the now much noted vessel. After having been thoroughly fumigated, scraped and washed at quarantine, she went to a dock in Brooklyn ; while there a clerk who occasionally visited her was taken with yellow fever and died. This shows at least that fumigation is not at all reliable, and I find that there is a growing skepticism about it. Indeed the only certain thing is fire. Next to it we must first try fumigation, hoping by this method to kill the germ—if it is a germ ; then, after this, we must try baking, in hopes that in this way we may decompose or drive off the infecting gas—if it is a gas. Mortals can do no more in that line at present.

New York has generally been thought to have plenty of hospitals but there is to be, in fact now is, one more. The Marine Hospital Service has established one on Bedloe's Island, an island in the harbor about a mile below the Battery. It will accommodate nearly one hundred and fifty patients and will soon be enlarged. The seamen treated by this service had heretofore been placed in the various city hospitals ; but it is the policy of the service—and a wise one—to treat its own patients, hence the present institution. It will prevent the dangers of any more yellow fever cases being carried to the city and be of much value in that respect. With the perfectly pure air (and plenty of it) which sweeps across the island there will be an opportunity also to learn whether Lister's method is not a superfluity under perfectly salubrious conditions. I am quite confident myself that such a conclusion will be reached. Much valuable work by the way, both medical and surgical, may be expected of this service in the course of a few years, for it is, as far as statistics are concerned, a vast hospital treating from twenty to thirty thousand patients a year, and these statistics are to be carefully worked up.

In visiting some hospitals a short time ago I was quite struck by the discrepancies in the treatment of pleurisy-with-effusion. In one hospital the use of the aspirator is absolutely forbidden no matter how long the effusion remains and provided of course that it does not become purulent. The dictum is that the third aspiration always causes empyæma. The treatment employed was that of Niemeyer : blisters, diuretics, tonics, possibly cathartics. A short time before, at another hospital, I saw about forty ounces of serum removed from a chest in which the disease had lasted for three or four weeks. There was no dyspnœa, but the patient had a little fever, had no appetite and was in fact not doing well. The serum did not re-accumulate after its removal and the patient steadily convalesced. There was no doubt about the benefit of the operation. Although no one will deny the danger of aspiration in subacute pleurisy it is not safe to dogmatize.

I fear I have put but little of interest or value in this letter, but I beg you to consider the temperature, and allow me to finish rather abruptly with a prescription for spermatorrhœa :

| | | |
|---|---|---|
| Tr. humuli | 40 | C. C. |
| Tr. camph. | 30 | C. C. |
| Tr. opii. | 10 | C. C. |
| Syr. tolu, q. s. ad | 100 | C. C. |

M. Sig. 4. C. C. at night.

It was given me by Dr. W. C. W. Glazier, Asst.-Surgeon Marine Hospital Service, and has had much endorsement, some of it very strong and rather amusing. The sailor is very subject to the affliction for which this is prescribed, but in him the disease almost always yields to the above remedy. One sailor who returned cured said that it had such a magic effect upon himself that he had given it to his messmates, and had in fact treated and cured the whole crew during his voyage; thereby winning for himself much glory and depriving the proprietor of a "Museum of Anatomy" here to whom seamen very generally resort, of a number of generous fees.

I will add another prescription, much used amongst this class, for gonorrhœa:

| | | |
|---|---|---|
| Zinci sulpat. | 2 | Grms. |
| Bole Armenian | 4 | " |
| Morph. sulph. | | 5 " |
| Aquæ | 572 | C. C. |

M. Sig. Use as injection four or five times a day.

There is nothing that will, as a rule, lessen the urethral discharge with more rapidity than this, at least amongst the seamen, and their quota of this particular variety of pathological secretion is sufficiently large to make the evidence quite valuable.

